# Immunization Coverage Surveys and Linked Biomarker Serosurveys in Three Regions in Ethiopia

**DOI:** 10.1371/journal.pone.0149970

**Published:** 2016-03-02

**Authors:** Mark A. Travassos, Berhane Beyene, Zenaw Adam, James D. Campbell, Nigisti Mulholland, Seydou S. Diarra, Tassew Kassa, Lisa Oot, Jenny Sequeira, Mardi Reymann, William C. Blackwelder, Yukun Wu, Inna Ruslanova, Jaya Goswami, Samba O. Sow, Marcela F. Pasetti, Robert Steinglass, Amha Kebede, Myron M. Levine

**Affiliations:** 1 Center for Vaccine Development, University of Maryland School of Medicine, Baltimore, Maryland, United States of America; 2 Ethiopian Public Health Institute, Addis Ababa, Ethiopia; 3 JSI Research & Training Institute Inc., Arlington, Virginia, United States of America; 4 Department of Social Work, The Women’s Hospital, Melbourne, Australia; 5 Centre pour le Développement des Vaccins, Mali (CVD-Mali), Bamako, Mali; University of Cambridge, UNITED KINGDOM

## Abstract

**Objective:**

Demographic and health surveys, immunization coverage surveys and administrative data often divergently estimate vaccination coverage, which hinders pinpointing districts where immunization services require strengthening. We assayed vaccination coverage in three regions in Ethiopia by coverage surveys and linked serosurveys.

**Methods:**

Households with children aged 12–23 (N = 300) or 6–8 months (N = 100) in each of three districts (woredas) were randomly selected for immunization coverage surveys (inspection of vaccination cards and immunization clinic records and maternal recall) and linked serosurveys. IgG-ELISA serologic biomarkers included tetanus antitoxin ≥ 0.15 IU/ml in toddlers (receipt of tetanus toxoid) and *Haemophilus influenzae* type b (Hib) anti-capsular titers ≥ 1.0 mcg/ml in infants (timely receipt of Hib vaccine).

**Findings:**

Coverage surveys enrolled 1,181 children across three woredas; 1,023 (87%) also enrolled in linked serosurveys. Administrative data over-estimated coverage compared to surveys, while maternal recall was unreliable. Serologic biomarkers documented a hierarchy among the districts. Biomarker measurement in infants provided insight on timeliness of vaccination not deducible from toddler results.

**Conclusion:**

Neither administrative projections, vaccination card or EPI register inspections, nor parental recall, substitute for objective serological biomarker measurement. Including infants in serosurveys informs on vaccination timeliness.

## Introduction

Given the contribution of infant immunization to plummeting young child mortality during the past 15 years[1,2], Gavi- the Vaccine Alliance, the Advanced Market Commitment and other donors have supported the introduction of costly new vaccines such as *Haemophilus influenzae* type b [Hib] conjugate, pneumococcal conjugates and rotavirus vaccines into the Expanded Program on Immunization (EPI) in developing countries [3]. Gavi also strengthens immunization services infrastructure based on a reward system for countries that measurably increase the number of children who receive three doses of diphtheria toxoid/tetanus toxoid/whole cell pertussis vaccine (DTP3 coverage) [3]. Ethiopia’s EPI, launched in 1980, administers BCG and oral polio vaccine (OPV) at birth; OPV, pentavalent vaccine (DTP, Hib conjugate and hepatitis B virus vaccine), and pneumococcal conjugate at ages six, 10 and 14 weeks; and measles vaccine at nine months. Daunting challenges confront the Ethiopian EPI as it grapples to deliver these vaccines to all infants in a timely way in a largely rural population that is sparsely dispersed in mountainous regions and often nomadic in arid areas [4]. Frustratingly, data from sources that should pinpoint districts needing improved immunization services are often starkly conflicting. For example, the Ethiopian national DTP3 coverage in 2010 based on official administrative estimates (number of vaccine doses administered by EPI to the target population divided by the number of target subjects [from census data]) was 86% [5]. In contrast, World Health Organization / United Nations Children’s Fund (WHO/UNICEF) joint reporting estimated 2010 DTP3 coverage at 63% [6,7], and a nationwide Demographic and Health Survey estimated only 37% DTP3 coverage based on sampling vaccination cards and parental recall [8].

With proper sampling and questionnaires, cluster surveys estimate the proportion of children who have received a particular vaccine [9–11]. However, since such surveys cannot indicate the quality of the vaccines administered nor can they confirm that a child given high-quality vaccine actually mounted an adequate immune response indicating protection, some have referred what coverage surveys measure as “coverage” and what biomarker surveys measure as “effective coverage” [11,12]. For these reasons, serosurveys that measure objective biomarkers performed concomitantly with immunization coverage cluster surveys are complementary tools to assess the performance of immunization services [11,13].

Serological biomarkers selected with respect to age, titer cut-offs and epidemiological facts can gauge immunization services’ effectiveness and timeliness. For example, tetanus antitoxin in toddlers derive only from immunization [14,15]. Whereas Hib anti-capsular polysaccharide [polyribosyl ribitol phosphate (PRP)] in toddlers may have derived from either infection with Hib or cross reacting bacteria, a high titer (≥ 1.0 mcg/ml) of anti-PRP in infants age 6–8 months denotes recent immunization rather than maternal transfer or infection-derived origin and also connotes durable protection [16–18]. Measuring specific antibodies also helps evaluate the integrity of the cold chain that underpins immunization services, since most vaccines must be assiduously maintained in the cold chain, lest they lose potency [15]. Live virus vaccines can be adversely affected by elevated temperatures, while protein-based vaccines can denature if inadvertently frozen [19]. Serosurvey biomarkers estimate objectively the prevalence of immunized (*i*.*e*., protected) children, irrespective of the prevalence of inoculated children (*i*.*e*., to whom vaccine was administered).

We linked serosurveys to immunization coverage surveys to measure the proportion of children protected against two pentavalent vaccine-preventable diseases (tetanus and invasive Hib) in three regions in Ethiopia [20]. Whereas coverage surveys typically focus on 12–23 month olds [21], we also sampled 6–8 month olds to assess the timeliness of infant immunization [22–24]. Overall, serologic biomarker measurements documented a hierarchy among the woredas, with Hintalo Wajerate (Tigray Region) showing the highest coverage. We found that objective serological biomarker measurements were not adequately estimated by administrative projections, vaccination card or EPI register inspections, or parental recall.

## Materials and Methods

In each of three administrative districts (woredas), 400 hundred children (N = 1200 total) were randomly selected to participate in immunization coverage surveys and accompanying serosurveys, from February to April 2013, lasting between 12 to 20 days in each woreda [20]. The selected districts, some of which had recent outbreaks of vaccine-preventable disease, included: Hintalo Wajerate woreda in Tigray Region (primarily agrarian with high administrative estimates of vaccine coverage); Arbegona in Southern Nations, Nationalities, and Peoples’ Region (“SNNPR”) (primarily agrarian, with lower administrative estimates of vaccine coverage); and Assaieta in Afar Region (pastoralist area with nomadic clans and low administrative estimates of vaccine coverage). Each woreda was selected by Ethiopian EPI and JSI Research & Training Institute, Inc. (JSI) to inform a Federal Ministry of Health (FMOH) evidence-based decision on how to pursue nationwide universal child immunization. The coverage survey sample per woreda was 300 toddlers aged 12–23 months and 100 infants aged 6–8 months. The serosurvey enrollment target was 60% of coverage survey enrollment.

The serosurvey protocol and consent procedure were approved by the Ethiopian National Research Ethics Review Committee and the University of Maryland, Baltimore Institutional Review Board. Written informed consent was obtained from parents or caretakers of each child enrolled in the serosurvey. Informed consent was documented by the use of a written consent form approved by the IRBs and signed or thumb printed by the parent or caretaker. Participation in the immunization coverage survey, a routine FMOH public health endeavor, did not require informed consent.

### Coverage surveys

Data were obtained by “JSI-type” coverage surveys that examine vaccination cards provided by parents/caretakers, record “parental recall” if cards were not available and peruse registers at health facilities where immunizations are administered to identify records of children whose parents claimed that they had been vaccinated but were unable to exhibit immunization cards [20].

### Antibody biomarker measurements

Serum IgG antibodies to vaccine antigens measured by ELISA served as biomarkers of acquisition of protective titers from vaccination or from wild type infection [22,25]. Protective titers of tetanus antitoxin in toddlers age 12–23 months denote successful immunization with pentavalent vaccine, which contains tetanus toxoid. A cut-off of ≥ 0.15 IU/ml, which correlates well with toxin neutralization [26], was the biomarker used for denoting subjects immunized with tetanus toxoid [25,27–30].

In infants age 6–8 months, a high (≥ 1.0 mcg/ml) titer of serum Hib IgG anti-PRP constitutes a biomarker that the infant received pentavalent vaccine in an age range approximating the recommended EPI schedule [22]. The anti-PRP biomarker is less useful in toddlers because by that age antibodies may also derive from natural exposure to Hib or similar bacteria [17].

### Statistics

A coverage survey child was considered immunized if she/he received three pentavalent vaccine doses via the routine EPI or via outreach or supplemental immunization activities. Results were compared against government administrative estimates of regional vaccine coverage (Health Sector Development Program (HSDP) IV. Woreda Based Health Sector Annual Core Plan); the administrative estimates were treated as constants in this analysis, since no measures of uncertainty were provided with them. The planned sample sizes of 300 toddlers aged 12–23 months and 100 infants aged 6–8 months per woreda for the coverage survey were the largest considered feasible. Assuming a design effect of 2 and 60% of the sample in the serosurvey, the width of exact two-sided 95% confidence intervals (CI’s) for the proportion of 12–23 month olds protected would be 21.5% and 17.4% for observed proportions of 50% and 80%, respectively; the power to show a significant difference from an administrative estimate of 90% would be approximately 0.82 for a true proportion protected of 80%.

Assuming the antibody biomarker gave the true status, positive predictive values (PPV) were calculated as the proportion of children positive by the antibody biomarker among children scored as positive by a coverage survey method of interest (vaccination card, scrutiny of immunization records or maternal recall) (true positives/[true positives + false positives]). Negative predictive values (NPV) were calculated as the proportion of children negative by the antibody biomarker among children scored as negative by a coverage survey method of interest (vaccination card, scrutiny of immunization records or maternal recall) (true negatives/[false negatives + true negatives]).

## Results

**Fig 1:**
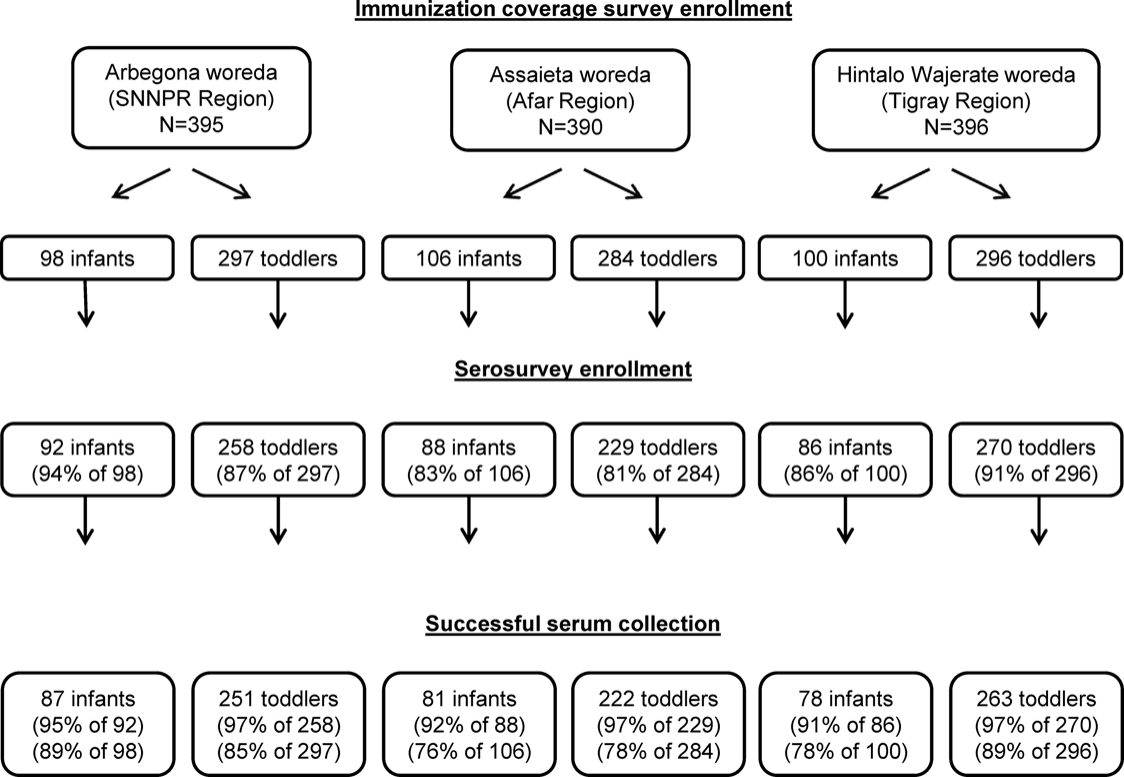
Consort diagram showing numbers of toddlers (age 12–23 months) and infants (age 6–8 months) enrolled into the immunization coverage survey in each in each woreda (administrative district), the number and percent subsequently enrolled into the serosurveys and the number and percent from whom blood was successfully obtained.

The JSI coverage surveys sampled 1,181 children in three woredas, of whom 1,023 (87%) were then enrolled in serosurveys (Fig 1); serum was collected from 96% of the serosurvey enrollees (N = 982). Serum was successfully collected from 222 toddlers from the Assaeita woreda, and serum quantity was sufficient to obtain antibody biomarker titers for 215 of these children; seven samples had inadequate serum volume to perform antibody measurements. Data in Table 1 display results for the 982 children who participated in both coverage survey and the serosurvey and yielded serum specimens.

**Table 1:** Pentavalent Vaccine-3 Coverage in Serosurvey Participants by Each Coverage Estimation Method and Age Group (Toddlers 12–23 Months Old and Infants 6–8 Months Old).

Coverage Estimate Method	Hintalo Wajerate woreda, Tigray Region		Arbegona woreda, SNNPR Region		Assaieta woreda, Afar Region	
Total population (Children < 2 yrs. of age)	174,582 (11,522)		161,307 (10,211)		54,479 (2690)	
	**Pentavalent vaccine-3 coverage in toddlers 12–23 months of age**					
	**Percentage**	**Absolute**	**Percentage**	**Absolute**	**Percentage**	**Absolute**
**Administrative (2012)**	90%^p = 0.15^		80%^p<0.0001^		85%^p<0.0001^	
**JSI coverage survey** (Vacc. card + maternal recall + EPI register)	87%^p = 0.017^ (82%, 91%)	229/263	41%^p<0.0001^ (35%, 47%)	103/251	35%^p<0.0001^ (29%, 42%)	75/215
***Serosurvey*: tetanus antitoxin** **≥** **0.15 IU/ml**	93% (89%, 96%)	244/263	60% (54%, 66%)	151/251	53% (46%, 60%)	114/215
	**Pentavalent vaccine-3 coverage in infants 6–8 months of age**					
	**Percentage**	**Absolute**	**Percentage**	**Absolute**	**Percentage**	**Absolute**
**Administrative (2012)**	90%^p<0.0001^		80%^p<0.0001^		85%^p<0.0001^	
**JSI coverage survey** (Vacc. card + maternal recall + EPI register)	58%^p = 0.096^ (46%, 69%)	45/78	29%^p = 0.071^ (20%, 39%)	25/87	40%^p = 0.19^ (29%, 51%)	32/81
***Serosurvey*: Hib PRP antibody** **≥** **1.0 mcg/ml**	68% (56%, 78%)	53/78	41% (31%, 52%)	36/87	31% (21%, 42%)	25/81

Administrative coverage estimates were treated as constants. Each administrative coverage estimate was compared to the corresponding serosurvey result by two-sided exact binomial test.

Each coverage survey result was compared to the corresponding serosurvey result by McNemar’s test (exact two-sided p value). Two-sided exact 95% confidence intervals are reported.

### Pentavalent-3 coverage

Administrative immunization coverage estimates indicated that one year before the surveys (2012), 80–90% of toddlers had received three pentavalent vaccine doses. JSI coverage surveys showed lower estimates of pentavalent-3 immunization compared to the administrative data and revealed disparity among the woredas (Table 1), with Hintalo Wajerate having the highest estimated coverage. Compared to the JSI coverage survey method, serological measurement of protective tetanus antitoxin biomarkers increased the estimate of pentavalent-3 coverage, reaching 93% in Hintalo Wajerate, 60% in Arbegona and 53% in Assaieta (Table 1). Further analyses (*vide infra*) consistently corroborated that Hintalo Wajerate achieved the best coverage among the woredas, indicating the most effective immunization services.

Among infants age 6–8 months, the JSI surveys estimated low pentavalent-3 coverage. Serological biomarker estimates of vaccination coverage with pentavalent vaccine based on the anti-PRP biomarker showed higher coverage estimates than did JSI coverage surveys in Hintalo Wajerate and Arbegona but not in Assaieta (Table 1).

### Serologic results and source of coverage information

We compared the proportion of subjects in each woreda with protective tetanus antitoxin biomarkers (toddlers) or PRP antibody biomarkers (infants) and the proportion recorded as having received three doses of pentavalent vaccine based on vaccination cards, EPI register records, or maternal recall (Table 2) Striking differences were observed among the woredas. In Hintalo Wajerate, only 10% of toddlers lacked documentation of vaccination with pentavalent vaccine by card or EPI register record (Table 2). In contrast, > 50% of toddlers in the other two woredas lacked documented pentavalent-3 vaccination, leaving only maternal recall for pentavalent-3 information. Among toddlers in each woreda, the estimation of coverage based on documented vaccination (vaccination card or EPI registry record) was only slightly lower (4–11%) than the prevalence of protective tetanus antitoxin biomarkers. Maternal recall led to significantly lower estimates compared to documented vaccination in each of the woredas (48% vs 91%, 9% vs 75% and 14% versus 68%). Moreover, in each woreda, among the toddlers whose evidence of vaccination derived from maternal recall, the prevalence of protective serologic biomarkers was higher than maternal recall estimates of coverage (68% vs 48%, 41% vs 9% and 37% vs 14%). In all three woredas, estimates of pentavalent coverage by immunization card were lower than the prevalence of protective serologic biomarkers. In contrast, EPI register record estimates were similar to biomarker findings. Across all three woredas, immunization cards or EPI register results had a high positive predictive value for serologic protection, but a poor negative predictive value. Maternal recall had poor positive and negative predictive values.

**Table 2:** Pentavalent Vaccine-3 Coverage in Serosurvey Participants by Documented (Vaccination Card or EPI Record) or Undocumented (Parental Recall Only) Source of Information.

		Documented vaccination			Undocumented vaccination
	Total (N)	Card or EPI register record	Cards	EPI register Record	Parental recall
	**Hintalo Wajerate pentavalent vaccine-3 coverage in toddlers 12–23 months of age**				
Coverage by source of information*	263	91% (217/238)^p = 0.076^	91% (149/163)^p = 0.049^	91% (68/75)^p = 1^	48% (12/25)^p = 0.18^
*Serosurvey*: tetanus antitoxin ≥ 0.15 IU/ml	263	95% (227/238)	97% (158/163)	92% (69/75)	68% (17/25)
Positive predictive value		96.3% (209/217)	97.3% (145/149)	94.1% (64/68)	41.7% (5/12)
Negative predictive value		14.3% (3/21)	7.1% (1/14)	28.6% (2/7)	69.2% (9/13)
		**Arbegona pentavalent vaccine-3 coverage in toddlers 12–23 months of age**			
Coverage by source of information*	251	75% (91/122)^p = 0.36^	54% (13/24)^p = 0.012^	80% (78/98)^p = 0.86^	9% (12/129)^p<0.0001^
*Serosurvey*: tetanus antitoxin ≥ 0.15 IU/ml	251	80% (98/122)	92% (22/24)	78% (76/98)	41% (53/129)
Positive predictive value		80.2% (73/91)	92.3% (12/13)	78.2% (61/78)	41.7% (5/12)
Negative predictive value		19.4% (6/31)	9.1% (1/11)	25.0% (5/20)	68.4% (80/117)
		**Assaieta pentavalent vaccine-3 coverage in toddlers 12–23 months of age**			
Coverage by source of Information*	215	68% (57/84)^p = 0.064^	68% (50/74)^p = 0.035^	70% (7/10)^p = 1^	14% (18/131)^p<0.0001^
*Serosurvey*: tetanus antitoxin ≥ 0.15 IU/ml	215	79% (66/84)	80% (59/74)	70% (7/10)	37% (48/131)
Positive predictive value		91.2% (52/57)	94.0% (47/50)	71.4% (5/7)	33.3% (6/18)
Negative predictive value		48.1% (13/27)	50% (12/24)	33.3% (1/3)	78.8% (89/113)
		**Hintalo Wajerate pentavalent vaccine-3 coverage in infants 6–8 months of age**			
Coverage by source of information*	78	61% (43/71)^p = 0.14^	60% (35/58)^p = 0.12^	62% (8/13)^p = 1^	29% (2/7)^p = 1^
*Serosurvey*: Hib PRP antibody ≥ 1.0 mcg/ml	78	70% (50/71)	73% (42/58)	62% (8/13)	43% (3/7)
Positive predictive value		95.3% (41/43)	97.1% (34/35)	87.5% (7/8)	100.0% (1/1)
Negative predictive value		35.7% (10/28)	34.8% (8/23)	40.0% (2/5)	80.0% (4/5)
		**Arbegona pentavalent vaccine-3 coverage in infants 6–8 months of age**			
Coverage by source of information*	87	50% (24/48)^p = 1^	41% (9/22)^p = 0.73^	58% (15/26)^p = 1^	3% (1/39)^p = 0.002^
*Serosurvey*: Hib PRP antibody ≥ 1.0 mcg/ml	87	52% (25/48)	50% (11/22)	54% (14/26)	28% (11/39)
Positive predictive value		91.7% (22/24)	100.0% (9/9)	86.7% (13/15)	100.0% (1/1)
Negative predictive value		29.2% (7/24)	30.8% (4/13)	27.3% (3/11)	73.7% (28/38)
		**Assaieta pentavalent vaccine-3 coverage in infants 6–8 months of age**			
Coverage by source of information*	81	83% (30/36)^p = 0.001^	83% (30/36)^p = 0.001^	0	4% (2/45)^p = 0.031^
*Serosurvey*: Hib PRP antibody ≥ 1.0 mcg/ml	81	47% (17/36)	47% (17/36)	0	18% (8/45)
Positive predictive value		93.3% (28/30)	93.3% (28/30)	-	100.0% (2/2)
Negative predictive value		83.3% (5/6)	83.3% (5/6)	-	86.0% (37/43)

* p values compare source of vaccination history to serosurvey results by McNemar’s test (exact two-sided p-value)

Positive predictive values and negative predictive values were calculated based on antibody biomarkers as the gold standard.

In Hintalo Wajerate, there were too few infants to analyze for whom there was no documentation of pentavalent vaccination (7 of 78). In contrast, in Arbegona and Assaieta, 45% and 56% of infants, respectively, lacked documentation of pentavalent vaccination. In these two woredas the pattern in infants was the same as among toddlers. The estimated pentavalent-3 coverage based on maternal recall was much lower than coverage estimates based on documented vaccination (3% vs 50% and 4% vs 83%). Moreover, the prevalence of protective PRP antibody biomarkers was much higher than the maternal recall estimate of coverage (28% vs 3% and 18% vs 4%). Thus, even when interviewing mothers of infants (where vaccination events were not as distant as for mothers of toddlers) maternal recall was unreliable. Similar to toddlers, across all three woredas, immunization cards or EPI register results had a high positive predictive value, but a poor negative predictive value.

### Protective antibody biomarkers in relation to number of doses of vaccine

With one exception (Arbegona, two doses), there was a clear dose-response association between the prevalence of protective tetanus antitoxin biomarkers and geometric mean titer (GMT) and the number of pentavalent vaccine doses received (Table 3). A similar effect was seen with receipt of two pentavalent doses (88%, 94% and 67%) in toddlers. Among infants 6–8 months of age with documented receipt of three doses of pentavalent vaccine, the prevalence of protective anti-PRP biomarkers was high in Hintalo Wajerate (88%) but lower in Arbegona (58%) and Assaieta (53%).

**Table 3:** Proportion of Toddlers (Age 12–23 Months) and Infants (0–11 Months) with Protective Titers of Antibodies to Pentavalent Vaccine Antigens and Geometric Mean Titers (GMT), According to the Number of Documented Doses of Vaccine Received by the Child.

**Pentavalent coverage by dose and tetanus antitoxin titer in toddlers 12–23 months**		
**Hintalo Wajerate**		
**No. of doses of pentavalent vaccine received by the child (documented by vaccination card or EPI register)**	**GMT, tetanus antitoxin**	**Number (%) of toddlers with tetanus antitoxin titers > 0.15 IU/ml**
3 doses (217 toddlers)	0.95 IU/ml	96% (209/217)
2 doses (16 toddlers)	0.85 IU/ml	88% (14/16)
1 dose (5 toddlers)	0.75 IU/ml	80% (4/5)
**238 toddlers total**		**95% (227/238)**
**Arbegona**		
**No. of doses of pentavalent vaccine received by the child (documented by vaccination card or EPI register)**	**GMT, tetanus antitoxin**	**Number (%) of toddlers with tetanus antitoxin titers > 0.15 IU/ml**
3 doses (91 toddlers)	0.47 IU/ml	80% (73/91)
2 doses (18 toddlers)	0.60 IU/ml	94% (17/18)
1 dose (13 toddlers)	0.34 IU/ml	62% (8/13)
**122 toddlers total**		**80% (98/122)**
**Assaieta**		
**No. of doses of pentavalent vaccine received by the child (documented by vaccination card or EPI register)**	**GMT, tetanus antitoxin**	**Number (%) of toddlers with tetanus antitoxin titers > 0.15 IU/ml**
3 doses (57 toddlers)	0.89 IU/ml	91% (52/57)
2 doses (9 toddlers)	0.22 IU/ml	67% (6/9)
1 dose (15 toddlers)	0.04 IU/ml	40% (6/15)
**81 toddlers total**		**79% (64/81)**
**Pentavalent coverage by dose and Hib PRP titer in infants 6–8 months**		
**Hintalo Wajerate**		
**No. of doses of pentavalent vaccine received by the child (documented by vaccination card or EPI register)**	**GMT, Hib PRP**	**Number (%) of infants with Hib PRP antibody** **≥** **1.0 mcg/ml**
3 doses (43 infants)	5.50 mcg/ml	88% (38/43)
2 doses (15 infants)	0.85 mcg /ml	53% (8/15)
1 dose (12 infants)	0.16 mcg /ml	25% (3/12)
**70 infants total**		**70% (49/70)**
**Arbegona**		
**No. of doses of pentavalent vaccine received by the child (documented by vaccination card or EPI register)**	**GMT, Hib PRP**	**Number (%) of infants with Hib PRP antibody** **≥** **1.0 mcg/ml**
3 doses (24 infants)	1.51 mcg /ml	58% (14/24)
2 doses (12 infants)	1.60 mcg /ml	67% (8/12)
1 dose (9 infants)	0.36 mcg /ml	33% (3/9)
**45 infants total**		**56% (25/45)**
**Assaieta**		
**No. of doses of pentavalent vaccine received by the child (documented by vaccination card or EPI register)**	**GMT, Hib PRP**	**Number (%) of infants with Hib PRP antibody** **≥** **1.0 mcg/ml**
3 doses (30 infants)	0.92 mcg /ml	53% (16/30)
2 doses (1 toddler)	0.38 mcg /ml	0% (0/1)
1 dose (4 infants)	0.09 mcg /ml	0% (0/4)
**35 infants total**		**28% (16/35)**

## Discussion

Gavi and other international agencies are scrutinizing how countries monitor the effectiveness of their immunization services, provide credible data to justify the financial investments, and identify under-performing districts needing improvements. Given this, coupling serosurveys that quantitatively measure biomarkers of immunization (or of seroprotection) with immunization coverage surveys in three rural districts of Ethiopia has yielded revelations of broad implication and utility: 1) corroboration of the inaccuracy of administrative estimates of vaccine coverage, which over-estimate coverage where immunization services are weak, i.e., where improvements are most needed [31]; 2) the erroneousness of parental recall in the rural Ethiopian setting, lending credence to the notion that in certain populations recall data should not be relied upon for decision making [11,13,32]; and 3) scrutinizing EPI registers, in addition to inspecting immunization cards, improves coverage survey accuracy but increases workload and provides no information on the immunization status of children lacking any documentation.

The detection of toddlers having a protective tetanus antitoxin biomarker added an objective benchmark to the survey and EPI register data. This is one of the first serosurveys in a pediatric population that received DTP vaccine bundled within pentavalent vaccine. The serum IgG tetanus antitoxin biomarker in toddlers clearly differentiated the three woredas, with Hintalo Wajerate exhibiting the highest prevalence of pentavalent-immunized toddlers, followed by Arbegona and Assaieta. In all three woredas, toddlers who had documented receipt of 2–3 doses of pentavalent vaccine by immunization card or by EPI register record had a high prevalence of tetanus antitoxin biomarkers.

Importantly, by including Hib PRP antibody biomarker measurement in 6–8 month old infants, invaluable information was derived on the timeliness of immunization with pentavalent vaccine. It is critical to adhere closely to the EPI schedule to maximize prevention of pertussis and invasive Hib disease, since pertussis deaths cluster in the first few months of infancy [33] and in sub-Saharan Africa Hib peaks at age 6–7 months [34]. Ordinarily age 6–8 months represents the nadir for prevalence of PRP antibody titers ≥ 1.0 mcg/ml; thus, absent pentavalent immunization, few African infants this age exhibit this biomarker [17,35]. Even in Hintalo Wajerate, the prevalence of protective PRP titers in infants was only 68% and was only 41% and 31% in the other woredas (Table 1). The infant biomarker measurements indicate that immunizations are being delivered later than scheduled in all woredas [36]. Indeed, in two woredas most 6–8 month olds sampled were vulnerable to pentavalent-vaccine preventable diseases, in contrast to toddlers from the same populations.

Vaccination record-keeping in Arbegona and Assaieta was problematic. In Arbegona, many children were apparently being vaccinated without a record, since 41% of toddlers lacking vaccination records exhibited protective tetanus antitoxin biomarkers. This phenomenon has also been reported in Africa among mothers women post-delivery in whom the prevalence of a history of prenatal tetanus toxoid vaccination was lower than the prevalence of tetanus antitoxin biomarker [37]. These findings can prompt action to improve immunization services in Arbegona. In Assaieta, where a proportion of the population is nomadic, some vaccines (particularly measles) are delivered via supplemental mass campaigns during which vaccination cards are not generally given to caretakers. Failure to issue immunization cards and poor record keeping at EPI vaccination units and during mass immunization campaigns are well-recognized sources of error in trying to interpret immunization coverage [11,32].

The Ethiopian experience of conducting concurrent coverage surveys and biomarker serosurveys in the same populations, a pioneering public health approach in Ethiopia and only rarely undertaken elsewhere [12,13,38], posed substantial logistical and other challenges but provided critical insights into the effectiveness of local EPI programs. In all three regions experienced phlebotomists overcame the challenge of collecting venous blood from infants and toddlers in the field, allowing us to measure tetanus and PRP antibodies in serum, a gold standard. However, to expand the use of serosurveys in developing countries, alternatives to venous blood collection must be considered to enhance practicality and economy. Emerging technologies are providing solutions to accomplish this. For example, collecting dried whole blood spots on filter papers following finger stick entirely avoid the need for skilled pediatric phlebotomists [39,40], having to centrifuge blood under field conditions and having to keep sera frozen. Collecting oral fluid specimens (which contain crevicular fluid rich in IgG) allows testing for IgG tetanus antitoxin [25] and other vaccine antibodies [41–43], while entirely eliminating the need for use of sharps. Indeed, portable point-of-care devices that detect protective IgG antibody biomarkers of vaccination in oral fluid that correlate closely with serum antibody levels are undergoing field trials.

Serosurveys (the term is used broadly here to include surveys that collect oral fluids) that measure tetanus antitoxin in toddlers in different districts can allow public health authorities in developing countries to monitor objectively the proportion of toddlers that have received DPT or pentavalent vaccine. In this way, districts can be assessed comparatively for the quality of the immunization services serving their populations [12]. Including 6–8 month old infants in whom PRP antibody biomarkers are measured can provide insights on the timeliness of immunization with pentavalent vaccine. Under-performing districts can be identified and remedial interventions introduced, while districts with objective biomarker evidence of high vaccination coverage can be studied to try and identify why such districts stand out. Such serosurveys can be carried out with or without an accompanying immunization coverage survey.

## Supporting Information

S1 Dataset Coverage survey findings and serological biomarker measurements of study participants. (XLSX)
